# Complete Relaxation of All the Uterine Ligaments

**Published:** 1891-08

**Authors:** W. A. Crow

**Affiliations:** Atlanta, Ga.


### COMPLETE RELAXATION OF ALL THE UTERINE LIGAMENTS.

BY W. A. CROW, M D., ATLANTA, GA.

Miss Annie L., white, age 25, virgin, family history good, of an extremely nervous temperament. Her health was good up to four years ago, when she broke down from over-work at school. Since then she has suffered from extreme weakness, being able to take little or no active outdoor exercise. She was seen by Prof. Goodell at his office during the fall. He advised the "rest cure," and gave her a prescription of Bashann's mixt. with strychnia.

She came under my care about December 1st, 1890, on account of a severe pain in lower part of abdomen, with acute paroxysms of pain coming on every three or four days. These attacks were so severe that an opiate was imperative. On examination I found an acute inflammation of the uterus and appendages, including vagina and bladder.

The uterus, at my first examination, was strongly anteverted, but I could not use a speculum on account of the acute pain, so I used the hot water douch, followed by astringents and sedatives, until I could introduce a Sims speculum; then I found the uterus in a retroverted position, very much to my surprise. This I corrected, and packed the vagina with pledgets of antiseptic wool, saturated with boro-glycerine, with alum. This was removed in 24 hours, and the douch used. This packing was again replaced in from 12 to 24 hours from the time it was removed. At the end of two weeks the inflammation had entirely left; there was no soreness.

During the time of the treatment I noticed the lack of any regularity of position of the uterus. At one time it would be misplaced forwards, then backwards or to either side, as it happened to be. I did not use the sound while there was any traces of inflammation, but when that subsided the sound was used and showed the uterus normal in size, and completely moveable in position, with little or no resistance, and a tendency to remain in any position placed, except where from gravity it would drop down. Such being the case, I found pressures of no avail, and to keep up the tampons, while it would relieve the trouble for the time, promised to be an infinite job.

So I had almost made up my mind that the only practical and rational treatment for anything like a permanent relief, was to bring on a premature menopause by removal of the ovaries. This would cause the uterus to atrophy, and thereby lessen its size and weight, which would at least promise relief. But before resorting to this extreme measure, I determined to try electricity, Faradic current, as the inflammatory symptoms were entirely removed, thereby removing the only counter indication to electricity.

These treatments were given every third day at first, using intra-uterine electrode, and a broad electrode over the lower part of abdomen, patient in dorsal position. The current was never strong enough to cause pain, still I could feel a strong contraction of the muscles at the maximum intensity of the current. The treatment lasted about five minutes at first, then from eight to ten minutes. The uterus was held as near normal position as possible, and was followed by the wool tampons, so as to retain the normal position as near as I could.

These treatments proved very satisfactory, being followed by a sense of relief to the patient from the beginning. At the end of a month I found very little misplacement on leaving the tampons out between the treatments, which were only given once a week after six weeks. She was discharged in March very much improved in general health, being entirely relieved of the pelvic pains and weakness, the uterus remaining in an approximately normal position.

